# ﻿A taxonomic revision of the freshwater crab genus *Parvuspotamon* Dai & Bo, 1994 (Decapoda, Brachyura, Potamidae), with descriptions of a new genus and two new species

**DOI:** 10.3897/zookeys.1183.109948

**Published:** 2023-10-26

**Authors:** Boyang Shi, Da Pan, Hongying Sun

**Affiliations:** 1 Jiangsu Key Laboratory for Biodiversity and Biotechnology, College of Life Sciences, Nanjing Normal University, Nanjing, 210023, China Nanjing Normal University Nanjing China

**Keywords:** China, Crustacea, new combination, Potamiscinae, taxonomy, Yunnan

## Abstract

The taxonomy of the potamid crab genus *Parvuspotamon* Dai & Bo, 1994, with two species native to Yunnan province of southwest China, is revised based on morphological and molecular data. In order to stabilise the taxonomy of these species (and the genus), two separate genera are hereby designated: *Parvuspotamon* and *Songpotamon***gen. nov.** While *Parvuspotamon* is restricted to *P.yuxiense* Dai & Bo, 1994, as a monotypic genus, *P.dixuense* Naruse, Chia & Zhou, 2018, is transferred to a new genus, *Songpotamon***gen. nov.** In addition, two new species of *Songpotamon***gen. nov.** are described herein: *S.funingense***sp. nov.** and *S.malipoense***sp. nov.***Songpotamon***gen. nov.** morphologically most resembles *Parvuspotamon* and *Chinapotamon* Dai & Naiyanetr, 1994, but can be distinguished by the combination of characters in the carapace, third maxilliped, thoracic sternites, and male first gonopod. The genetic data derived from the mitochondrial 16S rDNA also supports the monophyly of these new taxa.

## ﻿Introduction

The Yunnan Province of southwest China is noted for harbouring an exceptional number of freshwater species with a high level of endemism. This is widely recognised to be a direct consequence of the geological history of this mountainous region, e.g., orogenic processes and/or past climatic changes (Myers et al. 2000; Li et al. 2015; Antonelli et al. 2018; Rahbek et al. 2019; Pan et al. 2022). This diversity in landscape should have offered sufficient stability to the eco-environment, which not only supports the persistence of endemic species but also propels evolution and speciation (He and Jiang 2014; Atlas and Fu 2019; Ye et al. 2019; Wu et al. 2022).

Yunnan is a centre of diversification for Chinese freshwater crabs (Dai 1999; Cumberlidge et al. 2011). Although extensive research has been conducted on freshwater crabs in this region, the discovery of new genera and species still continues, and many new taxa have recently been revealed through morphological and molecular studies (Chu et al. 2017, 2018a, b; Naruse et al. 2018; Huang et al. 2020a, b; Wang et al. 2020; Zhang et al. 2020; Pan et al. 2021a, b, 2023; Tan et al. 2021; Shi et al. 2022). This is partly attributed to ongoing efforts focused on previously under-sampled areas.

**Figure 1.**
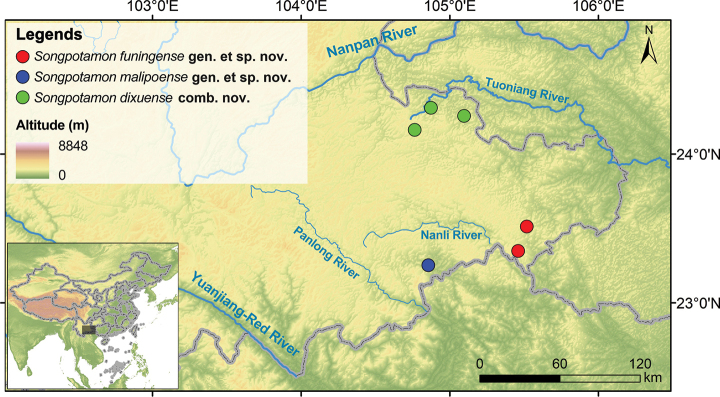
Map of southeast Yunnan showing the distribution of three species of *Songpotamon* gen. nov.

The genus *Parvuspotamon* Dai & Bo, 1994, was erected for its type species *Parvuspotamonyuxiense* Dai & Bo, 1994, which is currently only known from Yuxi City, Yunnan Province. Naruse et al. (2018) recently described *Parvuspotamondixuense* Naruse, Chia, & Zhou, 2018, from Yunnan, citing its morphological similarities with *Parvuspotamon*. Recent surveys in southwest China (Fig. 1) resulted in the collection of several freshwater crab specimens, which are similar to *P.dixuense*. Interestingly, these specimens are different from the type species of *Parvuspotamon*, i.e., *P.yuxiense*. After morphological comparisons and molecular phylogenetic inferences, we reveal that *Parvuspotamon* is polyphyletic and consists of two distinct clades. *Parvuspotamon* is therefore hereby revised, and a new genus, *Songpotamon* gen. nov., is established to accommodate *P.dixuense* and two new species, *Songpotamonfuningense* sp. nov. and *Songpotamonmalipoense* sp. nov.

## ﻿Materials and methods

### ﻿Crab collection

All individuals were collected from Yunnan Province. Specimens were preserved in 95% ethanol and were deposited at the Jiangsu Key Laboratory for Biodiversity and Biotechnology, College of Life Sciences, Nanjing Normal University, Nanjing, China (**NNU**) and the Institute of Zoology, the Chinese Academy of Sciences, Beijing, China (**CB**). The terminology is after Ng (1988), with changes as recommended by Dai (1999) and Davie et al. (2015). The abbreviations used are as follows: asl., above sea level; **G1**, male first gonopod; **G2**, male second gonopod.

### ﻿Phylogenetic analyses

DNA was extracted from gill or muscle tissues using the Trelief Animal Genomic DNA kit (Tsingke, Beijing, P.R. China) following the manufacturer’s protocol. A region of 490 base pairs (bp) of the 16S r DNA (16S) was amplified by polymerase chain reaction (PCR) using the primers 1471 and 1472 (Crandall and Fitzpatrick 1996). The PCR conditions included: initial denaturation at 95 °C for 3 min; 35 cycles of 30 s at 95 °C, 40 s at 50 °C, and 1 min at 72 °C; and a 7-min incubation at 72 °C. The following accession number were obtained from the GenBank: *Songpotamonfuningense* gen. et sp. nov., OR469050, OR469051, OR469054, OR469055, OR469057, and OR469058; *Songpotamonmalipoense* gen. et sp. nov., OR469052 and OR469053; *Songpotamondixuense* (Naruse, Chia & Zhou, 2018) comb. nov., OR469056 and OR544490; and *Parvuspotamonyuxiense*, OR469059 (also see type material sections).

All sequences were aligned using MAFFT v.7.215 (Katoh and Standley 2013), with the iterative refinement method G-INS-i (accurate alignment). The 16S dataset was compiled from both GenBank (*n* = 18) and newly generated data (*n* = 11). The Maximum-likelihood (ML) phylogenetic inference was performed using IQ-TREE v. 1.6.10 (Nguyen et al. 2015). The best substitution model was selected using MODELFINDER (Kalyaanamoorthy et al. 2017) as implemented in IQ-TREE v. 2. The Bayesian Inference (BI) analysis was conducted in MRBAYES v. 3.2.7a (Ronquist et al. 2012). Four chains were run simultaneously (three heated, one cold) for 10,000,000 generations, with tree space sampled every 1,000 generations. After a graphical analysis of the evolution of the likelihood scores, the first 250,000 generations were discarded as burn-in. The remaining trees were used to calculate the consensus tree. Acceptable convergence to the stationary distribution was checked by inspecting the posterior samples using the diagnostic software TRACER v. 1.7 (Rambaut et al. 2018). Effective sample sizes were > 200 for all parameters. The pairwise genetic distance among each species were calculated using MEGA X under the pairwise Kimura two-parameter (K2P) model (Kimura 1980; Kumar et al. 2018).

## ﻿Taxonomic account

**Family Potamidae Ortmann, 1896**

**Subfamily Potamiscinae Bott, 1970 (sensu Yeo and Ng 2004)**

### ﻿Genus Parvuspotamon

Taxon classificationAnimaliaDecapodaPotamidae

Dai & Bo, 1994

96446683-2328-5EB8-92B2-CB81DD77C366

Figs 2 , 3 , 4

#### Type species.

*Parvuspotamonyuxiense* Dai & Bo, 1994, by original designation.

#### Diagnosis.

**Figure 2.**
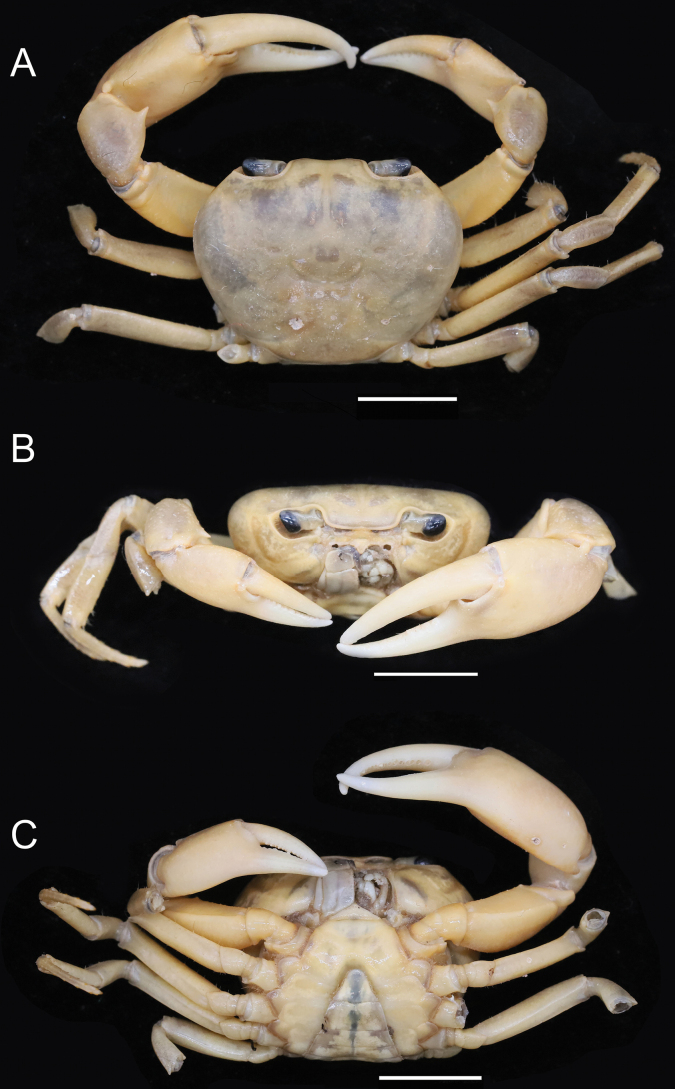
*Parvuspotamonyuxiense* Dai & Bo, 1994, ♂, 26.18 × 19.73 mm (NNU-3151-01) **A** overall dorsal view **B** overall frontal view **C** overall ventral view. Scale bars: 10 mm.

**Figure 3.**
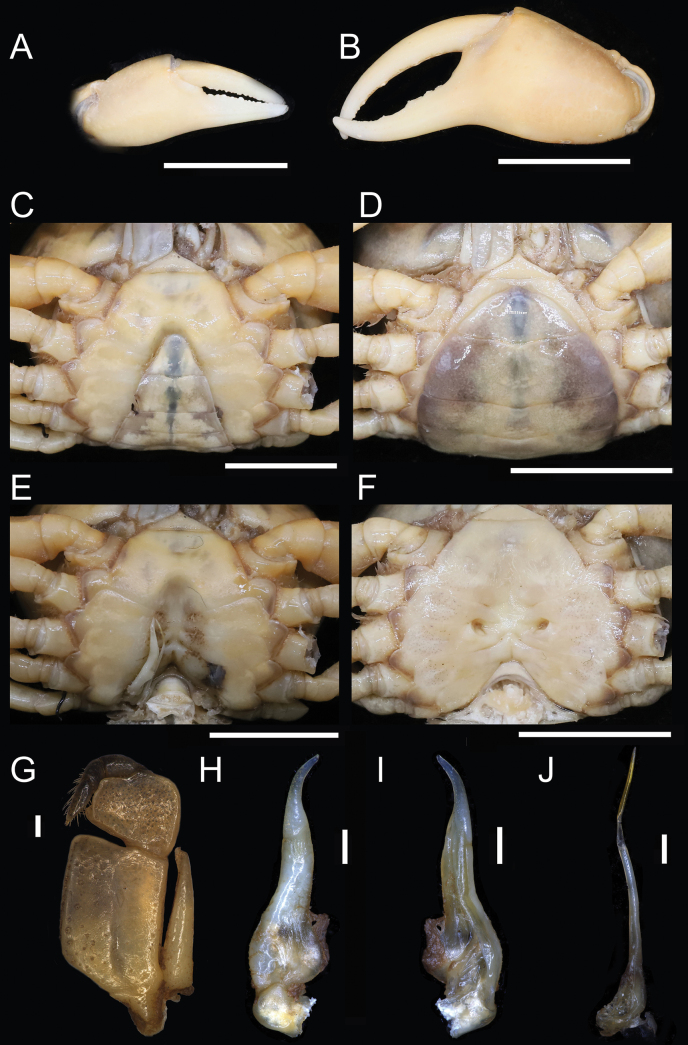
*Parvuspotamonyuxiense* Dai & Bo, 1994, ♂, 26.18 × 19.73 mm (NNU-3151-01) (**A–C, E, G–J**); ♀, 22.82 × 16.98 mm (NNU-3151-05) (**D, F**) **A** right chela **B** left chela **C** anterior thoracic sternum and pleon **D** pleon **E** thoracic sternum with right G1*in situ***F** thoracic sternum showing vulvae **G** left third maxilliped **H** dorsal view of left G1**I** ventral view of left G1**J** dorsal view of left G2. Scale bars: 10 mm (**A–F**); 1 mm (**G–J**).

**Figure 4.**
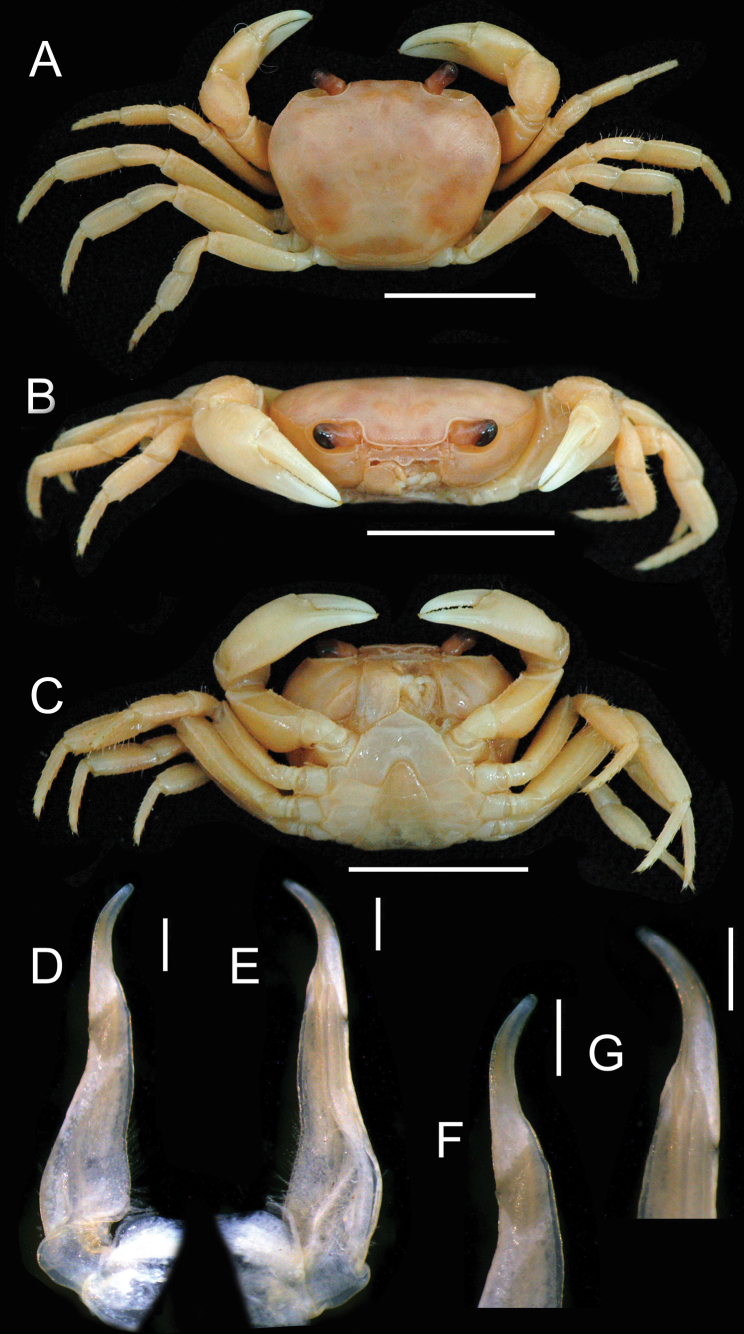
*Parvuspotamonyuxiense* Dai & Bo, 1994, holotype ♂, 15.7 × 12.9 mm (CB05138 YN 9091116A) **A** overall dorsal view **B** overall frontal view **C** overall ventral view **D** dorsal view of left G1**E** ventral view of left G1**F** dorsal view of left G1 distal portion **G** ventral view of left G1 distal portion. Scale bars: 10 mm (**A–C**); 1 mm (**D–G**).

Medium sized (adult carapace width 16–26 mm, *n* = 15). Carapace broader than long, ovate; dorsal surface convex, smooth, regions not clear; branchial regions swollen, smooth (Figs 2A, 4A). Epigastric cristae weakly developed, oblique, separated from each other by deep inverted Y-shaped groove; postorbital cristae low, indistinct, confluent with epigastric cristae (Figs 2A, 4A). External orbital angle bluntly triangular, outer margin and anterolateral margin of carapace confluent (Figs 2A, 4A). Anterolateral margin of carapace entire convex, smooth; posterolateral margins of carapace gently converging, smooth (Figs 2A, 4A). ﻿Epibranchial tooth indistinct (Figs 2A, 4A). Sub-orbital, sub-hepatic and pterygostomial regions smooth (Figs 2B, C, 4B, C). Antennular fossae slit-like in anterior view; median lobe of epistome posterior margin narrowly triangular (Figs 2B, 4B). Exopod of third maxilliped reaching beyond anterolateral corner of ischium, without flagellum (Fig. 3G). Thoracic sternites 3/4 in male completely fused (Figs 2C, 3C, E, 4C). Vulvae transversely ovate, widely located from each other, touching suture of sternites 5/6 (Fig. 3F). Male pleon broadly triangular (Figs 2C, 3C, 4C). G1 slender, reaching pleonal locking tubercle *in situ* (Figs 3E, H, I, 4D, E); subterminal segment stout, slightly sinuous (Figs 3H, I, 4D, E); terminal segment slender, relatively long, subconical, strongly sinuous, bent inwards, inner margin strongly concave, ~ 0.6× length of subterminal segment, without groove for G2 on ventral side, tip rounded, dorsal flap absent (Figs 3H, I, 4D–G). G2 longer than G1; terminal segment relatively long; subterminal segment ~ 1.5× length of terminal segment (Fig. 3J).

#### Remarks.

*Parvuspotamon* was previously known by two species, *P.yuxiense* (type species) and *P.dixuense*. The latter species was recently described by Naruse et al. (2018) based on the characters in the carapace and G1 terminal segment. Based on morphological and molecular data, *P.dixuense*, however, is transferred to *Songpotamon* gen. nov. since it possesses the generic characters of the new genus (see Remarks for the new genus). The present revision thus restricts *Parvuspotamon* only to the type species, i.e., *P.yuxiense*.

The morphological similarities between *Parvuspotamon* and *Songpotamon* gen. nov. notwithstanding, *Parvuspotamon* can easily be distinguished from *Songpotamon* gen. nov. by the characters in the carapace, vulvae and G1 (see Remarks for *Songpotamon* gen. nov.). *Parvuspotamon* is immediately distinguished from most of the remaining Chinese potamid genera by the combination of its medium body size (adult carapace width 16–26 mm), the strongly sinuous G1 terminal segment, and the absence of a flagellum on the exopod of the third maxilliped (Dai and Bo 1994; Dai 1999).

#### Geographic distribution.

*Parvuspotamon* is known only from Yunnan Province of southwest China.

### ﻿ Parvuspotamon yuxiense

Taxon classificationAnimaliaDecapodaPotamidae

﻿Dai & Bo, 1994

3862E8BA-3793-579A-8CB6-B1B410E8B8ED

Figs 2 , 3 , 4

#### Type material.

***Holotype*.** China • ﻿♂, 15.7 × 12.9 mm; Yunnan Province, Yuxi City, Xinping County, Gasa Township; Aug. 1990; CB05138 YN 9091116A.

#### ﻿Additional material.

China • 4 ♂, 26.18 × 19.73 mm (NNU-3151-01), 23.26 × 17.19 mm (NNU-3151-02), 22.68 × 17.02 mm (NNU-3151-03), 20.79 × 15.52 mm (NNU-3151-04), 3 ♀, 22.82 × 16.98 (NNU-3151-05), 21.13 × 16.47 (NNU-3151-06), 22.59 × 17.35 (NNU-3151-07); Yunnan Province, Yuxi City, Xinping County, Heshalak Village; 23.96°N, 101.45°E; altitude 955 m asl; 11 Apr. 2019; Boyang Shi, Xiyang Hao, Zewei Zhang, and Hongying Sun, leg. • 5 ♂, 24.94 × 18.94 mm (NNU-1513-01), 21.16 × 16.86 mm (NNU-1513-02), 22.02 × 16.06 mm (NNU-1513-03), 18.72 × 13.42 mm (NNU-1513-04), 16.58 × 11.98 mm (NNU-1513-05), 2 ♀, 22.54 × 16.63 mm (NNU-1513-06), 17.12 × 12.77 mm (NNU-1513-07); Yunnan Province, Yuxi City, Gasha Town; 24.02°N, 101.58°E; altitude 795 m asl; 15 Oct. 2015; Kelin Chu, Qiang Zhao, Pengfei Wang, and Hongying Sun leg.

#### Description.

Medium sized (adult carapace width 16–26 mm, *n* = 15). Carapace broader than long, ovate; dorsal surface convex both transversely and longitudinally, smooth, regions not clear; branchial regions swollen, smooth (Figs 2A, 4A). Postorbital and epigastric cristae confluent (Figs 2A, 4A); epigastric cristae weakly developed, oblique, separated by deep inverted Y-shaped groove (Figs 2A, 4A); postorbital cristae low, indistinct (Figs 2A, 4A). External orbital angle bluntly triangular, outer margin and anterolateral margin of carapace confluent (Figs 2A, 4A). Anterolateral margin of carapace entire convex, smooth; posterolateral margin gently concave, smooth, converging towards posterior carapace margin (Figs 2A, 4A). Epibranchial tooth indistinct (Figs 2A, 4A). Orbits large; supraorbital and infraorbital margins smooth; sub-orbital, sub-hepatic, and pterygostomial regions smooth (Figs 2B, C, 4B, C). Antennular fossae slit-like in anterior view; median lobe of epistome posterior margin narrowly triangular (Figs 2B, 4B). Third maxilliped with rhombus ischium; exopod reaching beyond anterolateral corner of ischium, without flagellum (Fig. 3G).

Chelipeds unequal (Figs 2A–C, 3A, B, 4A–C). Merus trigonal in cross section; margins weakly crenulated (Figs 2A, 4A). Carpus with sharp spine at inner-distal angle (Figs 2A, 4A). Major cheliped palm length ~ 1.3× height (Fig. 3B). Occlusal margin of fingers with several small teeth; distinct gape when closed (Fig. 3A, B).

Ambulatory legs not distinctly elongated, dactyli slender (Figs 2A, C, 4A, C); second pair longest, last pair shortest (Figs 2A, C, 4A, C). Outer surface of merus weakly rugose, dorsal margin weakly serrated, without subdistal tooth, length ~ 4.1× width (Figs 2A, 4A).

Male thoracic sternum smooth, weakly pitted; sternites 1/2 fused forming triangular structure; sternites 2/3 separated by deep but incomplete groove; sternites 3/4 completely fused; median longitudinal suture of sternites 7/8 deep (Figs 2C, 3C, E, 4C). Vulvae transversely ovate, widely located from each other, touching suture of sternites 5/6, posteromesial margin with low rim, opened obliquely upwards (Fig. 3F).

Male pleon broadly triangular; male telson relatively broad, lateral margins concave, width ~ 1.4× length; male pleonal somite 6 trapezoidal, broad, width ~ 2.3× length; somites 3–5 trapezoidal, gradually decreasing in width; somite 2 trapezoidal, reaching to bases of coxae of fourth ambulatory legs, thoracic sternite 8 not visible when pleon closed (Figs 2C, 3C, 4C). Female pleon ovate, covering most of thoracic sternum (Fig. 3D).

G1 slender, reaching pleonal locking tubercle *in situ*, with terminal and subterminal segments clearly demarcated (Figs 3E, H, I, 4D–G); subterminal segment stout, slightly sinuous (Figs 3H, I, 4D, E); terminal segment slender, relatively long, subconical, strongly sinuous, bent inwards, inner margin strongly concave, ~ 0.6× length of subterminal segment, without groove for G2 on ventral side, tip rounded, dorsal flap absent (Figs 3H, I, 4D–G). G2 longer than G1; terminal segment relatively long; subterminal segment ~ 1.5× length of terminal segment (Fig. 3J).

#### Colour in life.

Carapace and chelipeds are generally yellowish brown in mature individuals.

#### Habitat.

*Parvuspotamonyuxiense* can be found under rocks in hill streams at ~ 700–1000 m altitude.

#### Remarks.

*Parvuspotamonyuxiense* is the sole species of the genus and closely related to the species of *Songpotamon* gen. nov., and two species of *Tenuipotamon* Dai, 1990 (*Tenuipotamonyuxiense* Chen, 1993, and *Tenuipotamonxingpingense* Chen, 1993) that are known from Xinping County, Yuxi City of Yunnan Province. *Parvuspotamonyuxiense* can nevertheless be differentiated from *T.yuxiense* and *T.xingpingense* by the following characters: anterolateral margins of the carapace entire and smooth (vs cristate); and G1 terminal segment relatively less strongly curved, lacking a dorsal flap (vs more strongly curved, with a distinct dorsal flap) [cf. Chen 1993: figs 3 (4–6), 4 (4–6)]. On the other hand, *P.yuxiense* can be differentiated from the species of *Songpotamon* gen. nov. by the characters in the carapace, vulvae and G1 (see Remarks for *Songpotamon* gen. nov.).

#### Geographic distribution.

*Parvuspotamonyuxiense* is known only from the Yuxi City, Yunnan Province, southwest China.

### ﻿Genus Songpotamon gen. nov.

Taxon classificationAnimaliaDecapodaPotamidae

A7EDE512-5B51-55E0-B524-7DE472780CA5

https://zoobank.org/8CCC83D0-C234-4C34-AB6B-A3580AF601CD

**Figure 5.**
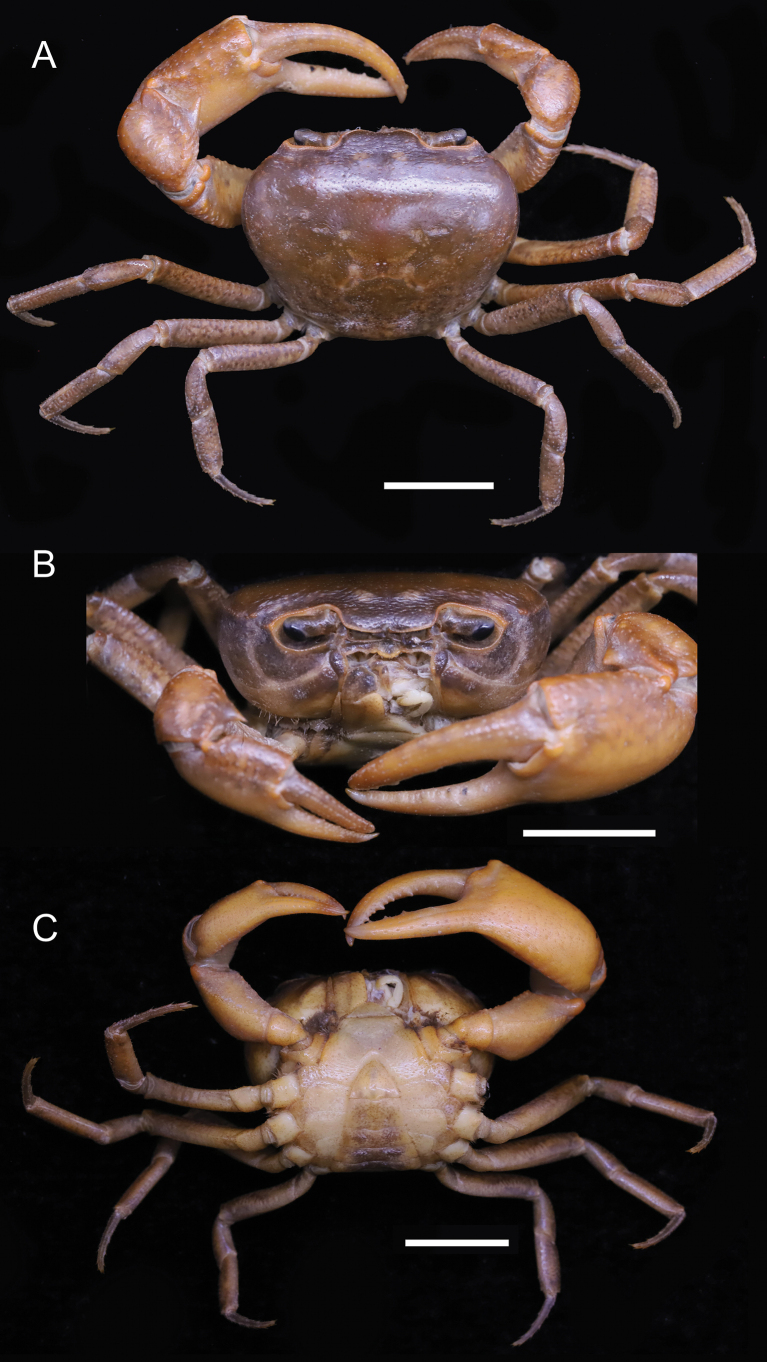
*Songpotamonfuningense* gen. et sp. nov., holotype ♂, 27.2 × 21.9 mm (NNU-167462-01) **A** overall dorsal view **B** frontal view of cephalothorax **C** overall ventral view. Scale bars: 10 mm.

**Figure 6.**
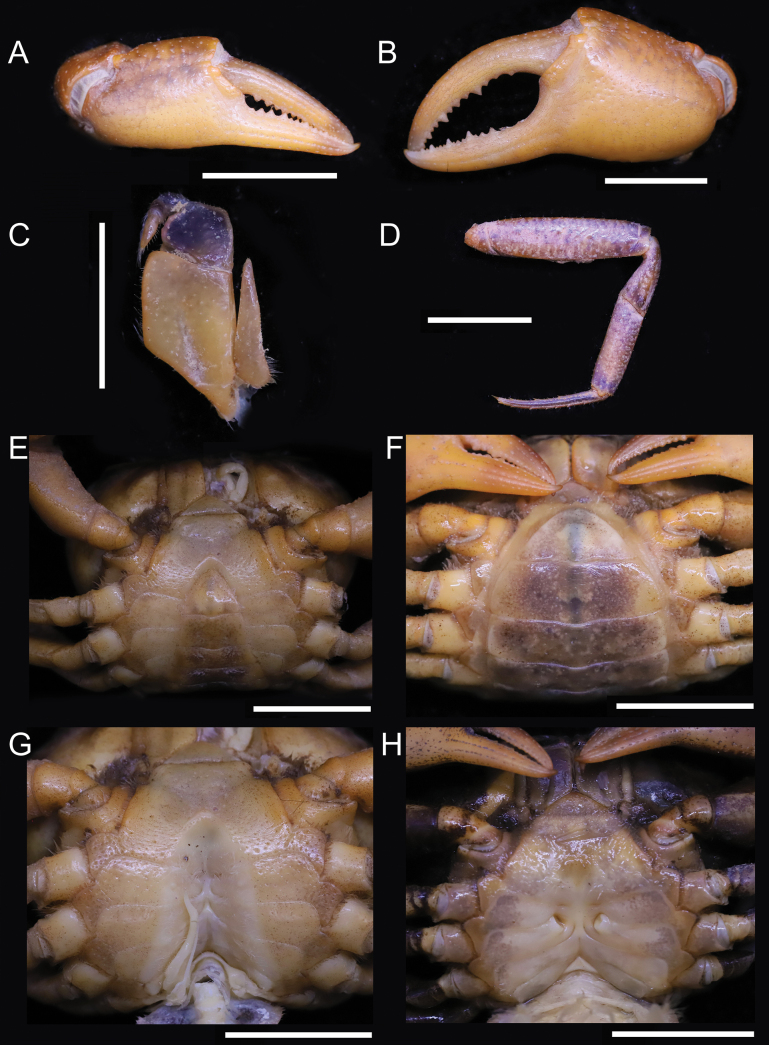
*Songpotamonfuningense* gen. et sp. nov., holotype ♂, 26.18 × 19.73 mm (NNU-3151-01) (**A–E, G**); paratype ♀, 22.6 × 18.0 mm (NNU-167462-05) (**F, H**) **A** right chela **B** left chela **C** left third maxilliped **D** right second ambulatory leg **E** anterior thoracic sternum and pleon **F** pleon **G** thoracic sternum with right G1*in situ***H** thoracic sternum showing vulvae. Scale bars: 10 mm.

**Figure 7.**
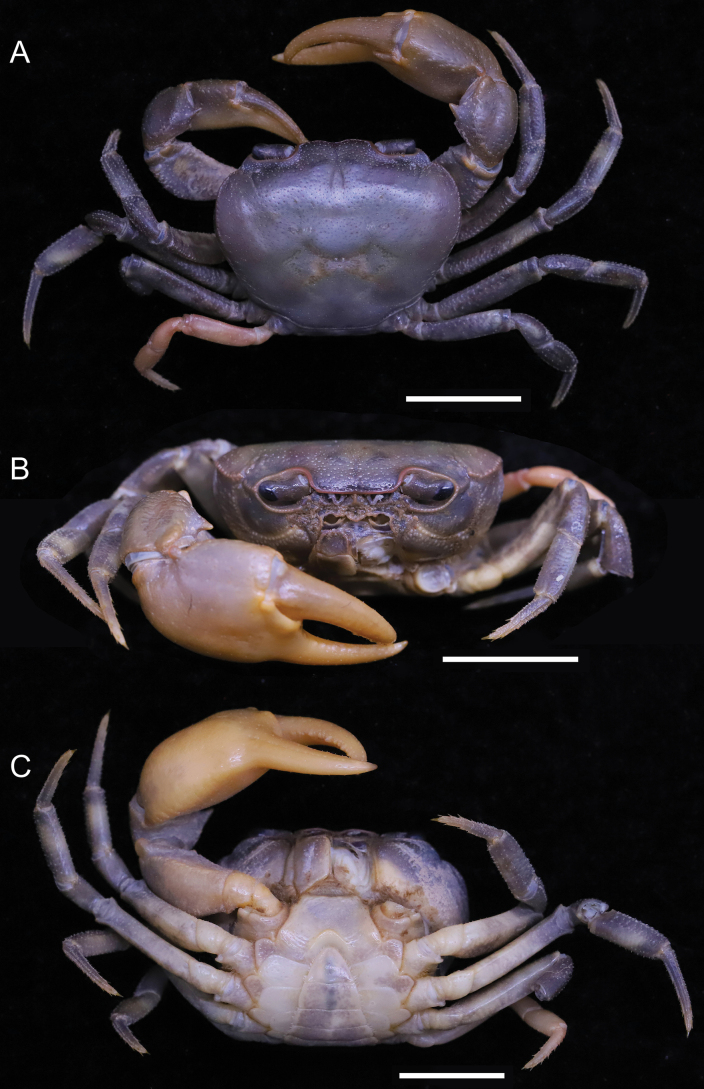
*Songpotamonmalipoense* gen. et sp. nov., holotype ♂, 21.7 × 16.8 mm (NNU-167444-01) **A** overall dorsal view **B** overall frontal view **C** overall ventral view. Scale bars: 10 mm.

**Figure 8.**
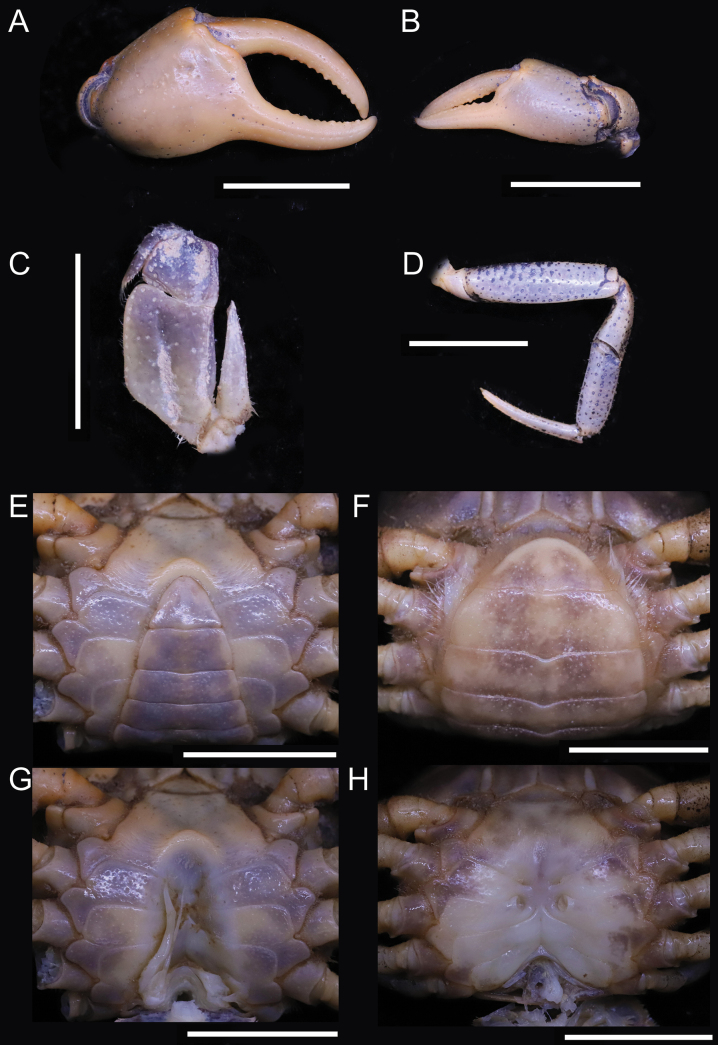
*Songpotamonmalipoense* gen. et sp. nov., holotype ♂, 21.7 × 16.8 mm (NNU-167444-01) (**A–E, G**); paratype ♀, 20.7 × 16.7 mm (NNU-167444-05) (**F, H**) **A** right chela **B** left chela **C** left third maxilliped **D** right second ambulatory leg **E** anterior thoracic sternum and pleon **F** pleon **G** thoracic sternum with right G1*in situ***H** thoracic sternum showing vulvae. Scale bars: 10 mm.

**Figure 9.**
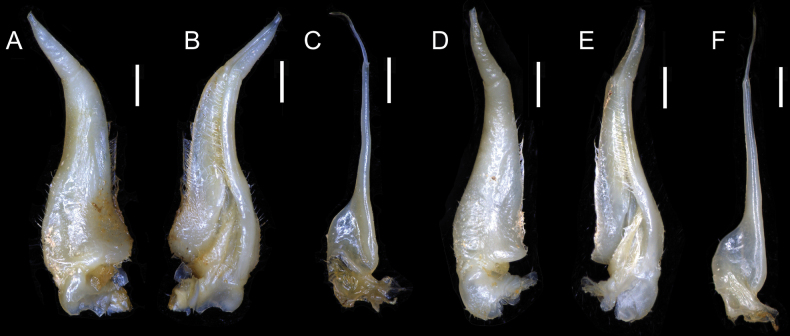
*Songpotamonfuningense* gen. et sp. nov., holotype ♂, 27.2 × 21.9 mm (NNU-167462-01) (**A–C**). *Songpotamonmalipoense* gen. et sp. nov., holotype ♂, 21.7 × 16.8 mm (NNU-167444-01) (**D–F**) **A, D** dorsal view of left G1**B, E** ventral view of left G1**C, F** dorsal view of left G2. Scale bars: 1 mm.

**Figure 10.**
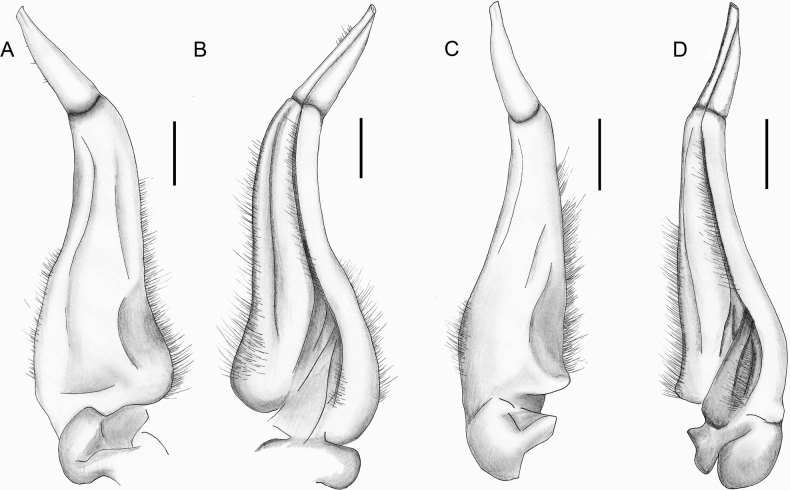
*Songpotamonfuningense* gen. et sp. nov., holotype ♂, 27.2 × 21.9 mm (NNU-167462-01) (**A, B**). *Songpotamonmalipoense* gen. et sp. nov., holotype ♂, 21.7 × 16.8 mm (NNU-167444-01) (**C, D**) **A, C** dorsal view of left G1**B, D** ventral view of left G1. Scale bars: 1 mm.

Figs 5 , 6 , 7 , 8 , 9 , 10

#### Type species.

*Songpotamonfuningense* sp. nov., by present designation.

#### Species included.

*Songpotamondixuense* (Naruse, Chia & Zhou, 2018), comb. nov., *Songpotamonfuningense* gen. et sp. nov., and *Songpotamonmalipoense* gen. et sp. nov.

#### Diagnosis.

Medium sized (adult carapace width 19–27 mm, *n* = 16). Carapace broader than long, ovate; dorsal surface convex, generally smooth, pitted, regions not clear; branchial regions swollen (Figs 5A, 7A). Postorbital and epigastric cristae not confluent, separated by shallow groove (Figs 5A, 7A). External orbital angle bluntly triangular, outer margin separated from anterolateral margin of carapace by shallow cleft (Figs 5A, 7A). Anterolateral margin of carapace convex (Figs 5A, 7A). Orbits large; supraorbital and infraorbital margins smooth (Figs 5B, 7B). Exopod of third maxilliped reaching beyond anterolateral corner of ischium, without flagellum (Figs 6C, 8C). Thoracic sternites 3/4 in male fused except for incomplete groove demarcating suture (Figs 5C, 6E, G, 7C, 8E, G). Vulvae transversely ovate, relatively closely located to each other, touching suture of sternites 5/6 (Figs 6H, 8H). Male pleon narrowly triangular (Figs 5C, 6E, 7C, 8E). G1 slender, almost reaching or reaching beyond pleonal locking tubercle *in situ* (Figs 6G, 8G, 9A, B, D, E, 10A–D); terminal segment slender, subconical, bent outwards, relatively short, ~ 0.4× length of subterminal segment, with distinct groove for G2 on ventral side, tip subtruncate, gently recurved upwards, dorsal flap absent (Figs 9A, B, D, E, 10A–D). G2 longer than G1; terminal segment relatively short; subterminal segment ~ 5× length of terminal segment (Fig. 9C, F).

#### Etymology.

The genus is named after the late Prof. Daxiang Song, a senior academician in the Chinese Academy of Sciences, in the honour of his immense contributions to Chinese invertebrate systematics; in arbitrary combination with the genus name *Potamon* Savigny, 1816. Gender of genus neuter.

#### Remarks.

*Songpotamon* gen. nov. is established for *S.dixuense* comb. nov. and two new species, *S.funingense* sp. nov. and *S.malipoense* sp. nov. *Songpotamondixuense* comb. nov. was previously in *Parvuspotamon*, but here transferred to *Songpotamon* gen. nov. because it possesses the key generic characters of the new genus, which includes, the medium body size (adult carapace width 19–27 mm), the third maxilliped exopod lacking a flagellum, the external orbital angle being bluntly triangular, the slender G1 shape, and the terminal segment being subconical and with a groove for G2 on the ventral side (cf. Naruse et al. 2018: figs 24–27).

*Songpotamon* gen. nov. can easily be distinguished from *Parvuspotamon* by the following characters: 1) carapace dorsal surface with scattered pits (Figs 5A, 7A; cf. Naruse et al. 2018: fig. 25A) (vs smooth; Figs 2A, 4A); 2) outer margin of the external orbital angle separated from the anterolateral margin of the carapace by the shallow cleft (Figs 5A, 7A; cf. Naruse et al. 2018: fig. 25A) (vs confluent with each other; Figs 2A, 4A); 3) vulvae relatively close located to each other (Figs 6H, 8H; cf. Naruse et al. 2018: fig. 27) (vs relatively widely located from each other; Fig. 3F); 4) G1 terminal segment relatively shorter, ~ 0.4× the length of the subterminal segment, with a truncated tip (Figs 9A, B, D, E, 10A–D; cf. Naruse et al. 2018: fig. 26A–D) (vs relatively longer, ~ 0.6× the length of the subterminal segment, with a rounded tip; Figs 3H, I, 4D–G); 5) G1 terminal segment bent outwards but with the tip gently recurved upwards, the inner margin being straight to gently curved (Figs 9A, B, D, E, 10A–D; cf. Naruse et al. 2018: fig. 26 A–D) (vs bent inwards, with the inner margin being strongly concave; Figs 3H, I, 4D–G); and 6) groove for G2 on the G1 terminal segment clearly visible in the ventral view (Figs 9 B, E, 10B, D; cf. Naruse et al. 2018: fig. 26A, B) (vs not visible; Figs 3I, 4E, G).

Furthermore, *Songpotamon* gen. nov. is most likely to be confused with *Chinapotamon* Dai & Naiyanetr, 1994, as both the genera have a very similar carapace physiognomy, and their G1 terminal segment is subconical, with the groove for G2 visible in the ventral view. The new genus, *Songpotamon* gen. nov., is nevertheless distinguished from *Chinapotamon* by the following characters: 1) carapace relatively high (Figs 5B, 7B) (vs relatively low); 2) ambulatory legs relatively stout (Figs 5A, C, 6D, 7A, C, 8D) (vs relatively slender); 3) anterolateral margin of the carapace being less convex (Figs 5A, 7A) (vs strongly convex); 4) third maxilliped exopod without flagellum (Figs 6C, 8C) (vs with well-developed flagellum); 5) thoracic sternites 3/4 with incomplete but distinct groove demarcating suture (Figs 5C, 6E, G, 7C, 8E, G) (vs groove demarcating suture absent); and 6) G1 terminal segment gently curved outwards (Figs 9A, B, D, E, 10A–D) (vs strongly bent outwards) (cf. Dai 1999: figs 42–47; Ng 2017: figs 2–4, 6–8; Zou et al. 2018: figs 2–6).

#### Geographic distribution.

*Songpotamon* gen. nov. is known from Wenshan Prefecture, eastern Yunnan Province, southwest China.

### ﻿ Songpotamon funingense

Taxon classificationAnimaliaDecapodaPotamidae

gen. et sp. nov.

91B34950-FA06-509E-9383-6E8BD63C6FFE

https://zoobank.org/8C1EEA1D-F6D9-4256-B0D4-1599AA753C99

Figs 5 , 6 , 9A–C , 10A, B

#### Type material.

***Holotype*.** China • ♂, 27.2 × 21.9 mm; Yunnan Province, Wenshan Prefecture, Funing County, Tianwan Township; 23.20°N, 104.87°E; altitude 880 m asl.; 22 Oct. 2020; Boyang Shi, Ruxiao Wang, and Hongying Sun leg.; GenBank: OR469050; NNU-167462-01.

***Paratype*.** China • ♂, 25.2 × 20.9 mm; same collection data as for holotype; GenBank: OR469051; NNU-167462-02 • ♂, 26.6 × 21.0 mm; same collection data as for holotype; GenBank: OR469057; NNU-167462-03 • ♂, 23.5 × 19.1 mm; same collection data as for holotype; GenBank: OR469058; NNU-167462-04 • ♀, 22.6 × 18.0 mm; same collection data as for holotype; NNU-167462-05 • ♀, 24.6 × 19.7 mm; same collection data as for holotype; NNU-167462-06 • ♂, 22.2 × 17.8 mm; Yunnan Province, Wenshan Prefecture, Funing County, Longbo Township; 23.31°N, 105.46°E; altitude 1611 m asl.; 24 Oct. 2020; Boyang Shi, Ruxiao Wang, and Hongying Sun leg.; GenBank: OR469054; NNU-167533-01 • ♂, 21.5 × 17.7 mm; same collection data as for NNU-167533-01; GenBank: OR469055; NNU-167533-02 • ♀, 20.8 × 16.4 mm; same collection data as for NNU-167533-01; NNU-167533-03.

#### Diagnosis.

Medium sized (adult carapace width 21–27 mm, *n* = 9). Carapace broader than long, ovate; dorsal surface convex, smooth, pitted, regions not clear; branchial regions swollen (Fig. 5A). Postorbital and epigastric cristae low, not confluent, separated by weak shallow groove (Fig. 5A); epigastric cristae weakly developed, straight, separated by shallow inverted Y-shaped groove; postorbital cristae low, rugose (Fig. 5A). External orbital angle bluntly triangular, outer margin convex, separated from anterolateral margin of carapace by shallow cleft (Fig. 5A). Anterolateral margin of carapace convex, generally smooth, weakly cristate; posterolateral margins gently converging, smooth (Fig. 5A). Orbits large; supraorbital and infraorbital margins smooth (Fig. 5B); sub-orbital, sub-hepatic and pterygostomial regions smooth or weakly rugose (Fig. 5B, C). Antennular fossae rectangular in anterior view; median lobe of epistome posterior margin low, rounded (Fig. 5B). Exopod of third maxilliped reaching beyond anterolateral corner of ischium, without flagellum (Fig. 6C). Thoracic sternites 3/4 in male fused except for relatively deep, incomplete groove demarcating suture (Figs 5C, 6E, G). Vulvae transversely ovate, closely located to each other, touching suture of sternites 5/6, opened obliquely ~ 45° upwards (Fig. 6H). Male pleon narrowly triangular; somite 6 relatively narrow, width ~ 2.4× as length (Fig. 5C). G1 slender, almost reaching pleonal locking tubercle *in situ* (Figs 6G, 9A, B, 10A, B); subterminal segment stout, sinuous, inner margin concave; terminal segment subconical, bent at ~ 45° outwards, relatively short, ~ 0.4× length of subterminal segment, with distinct groove for G2 on ventral side, tip subtruncate, recurved upwards (Figs 9A, B, 10A, B). G2 longer than G1; terminal segment relatively short; subterminal segment ~ 5× length of terminal segment (Fig. 9C).

#### Description.

Medium sized (adult carapace width 21–27 mm, *n* = 9). Carapace broader than long, ovate; dorsal surface convex transversely, longitudinally, smooth, pitted, regions not clear; branchial regions swollen, smooth (Fig. 5A). Postorbital and epigastric cristae inconspicuous, not confluent, separated by weakly shallow groove (Fig. 5A); epigastric cristae weakly developed, straight, separated by shallow Y-shaped groove; postorbital cristae low, rugose, reaching epibranchial tooth (Fig. 5A). Cervical groove indistinct (Fig. 5A). External orbital angle bluntly triangular, outer margin straight, with shallow cleft demarcating it from epibranchial tooth; epibranchial tooth weakly developed (Fig. 5A). Anterolateral margin convex, smooth, weakly cristate; posterolateral margin gently concave, smooth, converging towards posterior carapace margin (Fig. 5A). Orbits large; supraorbital and infraorbital margins smooth; sub-orbital, sub-hepatic, and pterygostomial regions relatively smooth or weakly rugose (Fig. 5B, C). Antennular fossae rectangular in anterior view; median lobe of epistome posterior margin low, rounded (Fig. 5B). Third maxilliped with rhombus ischium; exopod of third maxilliped reaching beyond anterolateral corner of ischium, without flagellum (Fig. 6C).

Chelipeds unequal (Figs 5A, C, 6A, B). Merus trigonal in cross section; margins crenulated (Fig. 5A, C). Carpus with sharp spine at inner-distal angle (Fig. 5A, C). Major cheliped palm length ~ 1.4× as height (Fig. 6B). Occlusal margin of fingers with sharp teeth; distinct gape when closed (Fig. 6B).

Ambulatory legs not distinctly elongated, dactyli slender (Figs 5A, C, 6D); second pair longest, last pair shortest (Fig. 5A, C). Outer surface of merus slightly rugose, dorsal margin weakly serrated, without subdistal tooth, length ~ 3.7× as width (Fig. 6D).

Male thoracic sternum generally smooth, weakly pitted; sternites 1/2 fused to form a triangular structure (Figs 5C, 6E, G); sternites 2/3 demarcated by horizontal groove; sternites 3/4 fused except for relatively deep, incomplete groove demarcating suture (Figs 5C, 6E, G); median longitudinal suture of sternites 7/8 deep (Fig. 6E, G). Vulvae transversely ovate, closely located to each other, touching suture of sternites 5/6, opened obliquely ~ 45° upwards, posteromesial margin with low raised rim (Fig. 6H).

Male pleon narrowly triangular; telson relatively broad, lateral margins slightly convex, width ~ 1.2× as length (Figs 5C, 6E); somite 6 broadly rectangular, width ~ 2.4× as length; suture between somites 6/7 sinuous; somites 3–5 trapezoidal, gradually decreasing in width; somite 2 trapezoidal, reaching to bases of coxae of fourth ambulatory legs; thoracic sternite 8 not visible when pleon closed (Figs 5C, 6E). Female pleon ovate, covering most of thoracic sternum (Fig. 6F).

G1 slender, almost reaching pleonal locking tubercle *in situ*, with terminal and subterminal segments clearly demarcated (Figs 6G, 9A, B, 10A, B); subterminal segment stout, sinuous, distal part prominently narrow, inner margin concave (Figs 9A, B, 10A, B); terminal segment slender, subconical, bent at ~ 45° outwards, relatively short, ~ 0.4× length of subterminal segment, outer margin slightly convex, inner margin straight, with distinct groove for G2 on ventral side, tip subtruncate, recurved upwards (Figs 9A, B, 10A, B). G2 longer than G1, terminal segment relatively short; subterminal segment ~ 5× length of terminal segment (Fig. 9C).

#### Etymology.

The species is named after Funing County, the type locality of the new species in the Yunnan Province of China.

#### Colour in life.

Carapace and chelipeds are generally bright orange to red with purplish brown ambulatory legs in mature individuals. Generally purplish brown all over with bright orange tips of the chelipeds in smaller individuals.

#### Habitat.

This new semi-terrestrial species digs and inhabits mud burrows close to small hill streams and seeps.

#### Remarks.

The new species most closely resembles *S.malipoense* gen. et sp. nov., in general carapace morphology, especially in possessing the relatively narrower male pleonal somite 6, the recurved tip of the G1 terminal segment, and the distinct and entire groove for the G2 on the ventral side of the G1 terminal segment. *Songpotamonfuningense* gen. et sp. nov., however, can be separated from *S.malipoense* gen. et sp. nov. by the following characters: 1) anterolateral margins of the carapace generally smooth (Fig. 5A) (vs with small granules; Fig. 7A); epigastric cristae straight in dorsal view and separated from each other by a shallow inverted Y-shaped groove (Fig. 5A) (vs oblique in dorsal view and separated from each other by a relatively deep inverted Y-shaped groove; Fig. 7A); male thoracic sternites 3/4 with relatively deep groove demarcating suture (Figs 5C, 6E, G) (vs relatively shallow; Figs 7C, 8E, G); vulvae opening obliquely ~ 45° upwards (Fig. 6H) (vs opening inwards; Fig. 9H); G1 subterminal segment relatively stouter, with the inner margin concave (Figs 9A, B, 10A, B) (vs relatively slenderer, with the inner margin almost straight; Figs 9D, E, 10C, D); and G1 terminal segment strongly bent at ~ 45° outwards (Figs 9A, B, 10A, B) (vs gently curved at ~ 30° outwards ; Figs 9D, E, 10C, D).

The new species is also morphologically similar to *S.dixuense* comb. nov. in the weakly developed epibranchial tooth, the relatively smooth sub-orbital, sub-hepatic, and pterygostomial regions, and the sinuous subterminal segment of the G1. *Songpotamonfuningense* gen. et sp. nov., however, can be distinguished from *S.dixuense* comb. nov. by the following characters: antennular fossae subrectangular (Fig. 5B) (vs slit-like); male pleonal somite 6 relatively narrower (Fig. 5C) (vs relatively broader); vulvae opening distinctly oblique ~ 45° upwards (Fig. 6H) (vs opening slightly oblique ~ 30° upwards); G1 subterminal segment relatively stouter (Figs 9A, B, 10A, B) (vs relatively slenderer); and G1 terminal segment relatively strongly bent at ~ 45° outwards (Figs 9A, B, 10A, B) (vs gently curved at ~ 30° outwards) (cf. Naruse et al. 2018: figs 24B, 26, 27).

#### Geographic distribution.

*Songpotamonfuningense* gen. et sp. nov. is known from Funing County, eastern Wenshan Prefecture, Yunnan Province, southwest China.

### ﻿ Songpotamon malipoense

Taxon classificationAnimaliaDecapodaPotamidae

gen. et sp. nov.

040D1417-5DB3-5B3C-8581-837161CDF30D

https://zoobank.org/6D3DB24F-9039-4DDB-A1C7-F0F46443826C

Figs 7 , 8 , 9D–F , 10C, D

#### Type material.

***Holotype*.** China • ♂, 21.7 × 16.8 mm; Yunnan Province, Wenshan Prefecture, Malipo County, Tiechang Township; 23.20°N, 104.83°E; altitude 864 m asl.; 24 Oct. 2020; Boyang Shi, Ruxiao Wang, and Hongying Sun leg.; GenBank: OR469052; NNU-167444-01.

***Paratype*.** China • ♂, 22.1 × 17.8 mm; same collection data as for holotype; GenBank: OR469053; NNU-167444-02 • ♂, 20.3 × 16.2 mm; same collection data as for holotype; NNU-167444-03 • ♂, 19.6 × 15.7 mm; same collection data as for holotype; NNU-167444-04 • ♀, 20.7 × 16.7 mm; same collection data as for holotype; NNU-167444-05 • ♀, 20.5 × 15.4 mm; same collection data as for holotype; NNU-167444-06 • ♀, 18.6 × 14.3 mm; same collection data as for holotype; NNU-167444-07.

#### Diagnosis.

Medium sized (adult carapace width 19–22 mm, *n* = 7). Carapace broader than long, ovate; dorsal surface convex, smooth, pitted, regions not clear; branchial regions swollen, smooth (Fig. 7A). Postorbital and epigastric cristae inconspicuous, not confluent, separated by shallow groove (Fig. 7A); epigastric cristae weakly developed, oblique, separated by deep inverted Y-shaped groove; postorbital cristae low, weakly rugose (Fig. 7A). External orbital angle bluntly triangular, outer margin convex, separated from anterolateral margin of carapace by shallow cleft (Fig. 7A). Anterolateral margin of carapace convex, cristate, granular; posterolateral margin straight, with multiple weakly oblique striae (Fig. 7A). Orbits large; sub-orbital regions smooth; sub-hepatic and pterygostomial regions with small, rounded granules (Fig. 7B, C). Antennular fossae semi-circular; median lobe of epistome posterior margin broadly triangular (Fig. 7B). Exopod of third maxilliped reaching beyond anterolateral corner of ischium, without flagellum (Fig. 8C). Thoracic sternites 3/4 in male fused except for relatively shallow, incomplete groove demarcating suture (Figs 7C, 8E, G). Vulvae transversely ovate, closely located to each other, touching suture of thoracic sternites 5/6, opened inwards (Fig. 8H). Male pleon narrowly triangular; somite 6 relatively narrow, width ~ 2.5× as length (Fig. 8E). G1 slender, reaching beyond pleonal locking tubercle up to suture between thoracic sternites 4/5 *in situ* (Figs 8G, 9D, E, 10C, D); subterminal segment relatively slender, gently sinuous, inner margin almost straight; terminal segment short, slender, subconical, relatively less strongly bent at ~ 30° outwards, ~ 0.4× length of subterminal segment, with distinct groove for G2 on ventral side, tip subtruncate, recurved upwards (Figs 9D, E, 10C, D). G2 longer than G1; terminal segment relatively short; subterminal segment ~ 5× length of terminal segment (Fig. 9F).

#### Description.

Medium sized (adult carapace width 19–22 mm, *n* = 7). Carapace broader than long, ovate; dorsal surface convex transversely, longitudinally, smooth, pitted, regions not clear; branchial region swollen, smooth (Fig. 7A). Postorbital and epigastric cristae inconspicuous, not confluent, separated by shallow groove; epigastric cristae weakly developed, oblique, separated by deep inverted Y-shaped groove; postorbital cristae low, weakly rugose, reaching epibranchial tooth (Fig. 7A). Cervical groove distinct, shallow (Fig. 7A). External orbital angle bluntly triangular, outer margin convex, with shallow cleft demarcating it from epibranchial tooth; epibranchial tooth weakly developed (Fig. 7A). Anterolateral margin of carapace convex, cristate, granular; posterolateral margin straight, with multiple weakly oblique striae, converging towards posterior carapace margin (Fig. 7A). Orbits large; supraorbital and infraorbital margins smooth; sub-orbital regions smooth, sub-hepatic and pterygostomial regions with small, rounded granules (Fig. 7B, C). Antennular fossae semi-circular in anterior view; median lobe of epistome posterior margin broadly triangular (Fig. 7B). Third maxilliped with subrectangular ischium; exopod of third maxilliped reaching beyond anterolateral corner of ischium, without flagellum (Fig. 8C).

Chelipeds unequal (Figs 7A, C, 8A, B). Merus trigonal in cross section; margins crenulated (Fig. 7A, C). Carpus with bluntly stout spine at inner-distal angle (Fig. 7A, C). Major cheliped palm length ~ 1.2× as height (Fig. 8A). Occlusal margin of fingers with rounded, blunt teeth; distinct gape when closed (Fig. 8A).

Ambulatory legs not elongated, slender dactyli (Figs 7A, C, 8D); second pair longest, last pair shortest (Fig. 7A, C). Outer surface of merus slightly rugose, dorsal margin weakly serrated, without subdistal tooth, length ~ 3.5× as width (Fig. 8D).

Male thoracic sternum generally smooth, weakly pitted; sternites 1/2 fused forming triangular structure (Figs 7C, 8E, G); sternites 2/3 demarcated by horizontal groove; sternites 3/4 fused except for relatively shallow, incomplete groove demarcating suture (Figs 7C, 8E, G); median longitudinal suture of sternites 7/8 deep (Fig. 8E, G). Vulvae transversely ovate, closely located to each other, touching suture of thoracic sternites 5/6, opened inwards, posteromesial margin with low raised rim (Fig. 8H).

Male pleon narrowly triangular; telson relatively broad, lateral margins slightly convex, width ~ 1.3× as length (Figs 7C, 8E); somite 6 broadly rectangular, width ~ 2.5× as length; suture between somites 6/7 sinuous; somites 3–5 trapezoidal, gradually decreasing in width; somite 2 trapezoidal, reaching to bases of coxae of fourth ambulatory legs; thoracic sternite 8 not visible when pleon closed (Figs 7C, 8E). Female pleon ovate, covering most of thoracic sternum (Fig. 8F).

G1 slender, reaching beyond pleonal locking tubercle up to suture between thoracic sternites 4/5 *in situ*, with terminal and subterminal segments clearly demarcated (Figs 8G, 9D, E, 10C, D); subterminal segment relatively slender, gently sinuous, distal part prominently narrow, inner margin almost straight (Figs 9D, E, 10C, D); terminal segment short, slender, subconical, relatively less strongly bent at ~ 30° outwards, ~ 0.4× length of subterminal segment, outer margin convex, inner margin convex, with distinct groove for G2 on ventral side, tip subtruncate, recurved upwards (Figs 9D, E, 10C, D). G2 longer than G1, terminal segment relatively short; subterminal segment ~ 5× length of terminal segment (Fig. 9F).

#### Etymology.

The species is named after Malipo County, the type locality of the new species in the Yunnan Province of China.

#### Colour in life.

The dorsal surface of the carapace is dark brown, with brighter chelae; the ventral surface is paler.

#### Habitat.

*Songpotamonmalipoense* gen. et sp. nov. is usually found hiding under rocks in small hill streams. Some large specimens have nevertheless been collected from deep mud burrows at the bank of hill streams, suggesting a semi-terrestrial lifestyle.

#### Remarks.

The new species superficially resembles *S.dixuense* comb. nov. in overall carapace physiognomy, especially in possessing the granular anterolateral margin of the carapace and the relatively slender G1. *Songpotamonmalipoense* gen. et sp. nov., however, can immediately be distinguished from *S.dixuense* comb. nov. by the following characters: 1) carapace dorsal surface glabrous, with dense pits (Fig. 7A) (vs with few short setae and scattered pits); 2) inverted Y-shaped groove between the epigastric cristae relatively deep (Fig. 7A) (vs relatively shallow); 3) chela generally smooth on the outer surface (Fig. 8A) (vs relatively rugose); 4) pterygostomial regions with prominent rounded granules (Fig. 7B) (vs relatively smooth); 5) antennular fossae rectangular in anterior view (Fig. 7B) (vs slit-like); 6) median tooth on the epistome posterior margin broadly triangular (Fig. 7B) (vs narrowly triangular); 7) male pleonal somite 6 relatively narrower (vs relatively broader); and 8) G1 with an almost straight inner margin of the subterminal segment (Fig. 9D, E, 10C, D) (vs with a gently concave inner margin) (cf. Naruse et al. 2018: figs 24A, B, 25B, 26A, C). Biogeographically, these two species are also isolated due to their occurrence in different drainages, with the new species in the Yuanjiang-Red River Basin and *S.dixuense* comb. nov. in the Pearl River Basin (Fig. 1). *Songpotamonmalipoense* gen. et sp. nov. need not be confused with *S.funingense* gen. et sp. nov. (see Remarks for the latter new species).

#### Geographic distribution.

*Songpotamonmalipoense* gen. et sp. nov. is known from Malipo County, southern Wenshan Prefecture, Yunnan Province, southwest China.

## ﻿Phylogenetic analysis and discussion

**Figure 11.**
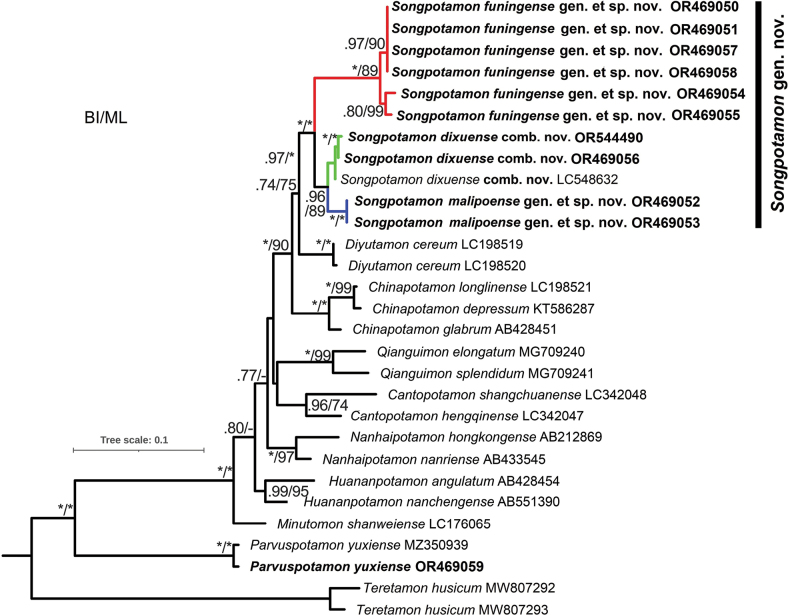
Maximum Likelihood phylogenetic tree based on 16S rDNA sequences. Support values at the nodes represent the > 50% posterior probabilities (PP) and bootstrap values (BV) for BI and ML (PP/BV), respectively. The new sequences are indicated in bold font. Asterisks indicate PP = 1.00 or BS = 100.

A total of 18 potamid species from 10 genera, including the new genus and two new species, and *P.yuxiense*, were used in the analysis. A 490 bp16S rDNA segment, excluding the variable regions, was aligned. ML and BI analyses resulted in congruent tree topologies with some minor differences in the terminal lineages. The phylogenetic results inferred that two new species and *S.dixuense* comb. nov. formed a well-supported monophyletic lineage (Fig. 11). *Parvuspotamonyuxiense* is situated at a basal position and is far away from the new genus, *Songpotamon* gen. nov., in the phylogenetic tree. *Songpotamon* gen. nov. is phylogenetically more closely related to *Diyutamon* Huang, Shih & Ng, 2017, and *Chinapotamon* than to *Parvuspotamon*, occurring in southeast Yunnan. These genera, however, are not only morphologically distinct but also characterised by different geographic distributions and habitats, i.e., *Diyutamon* colonised subterranean karst streams (Huang et al. 2017), and *Chinapotamon* occurred in both subterranean karst streams and hill streams (Dai and Naiyanetr 1994; Ng 2017; Zou et al. 2018).

The phylogenetic trees suggested that two new species of *Songpotamon* gen. nov. cluster with their congener *S.dixuense* comb. nov. *Songpotamonmalipoense* gen. et sp. nov. is close to *S.dixuense* comb. nov. (PP/BV = 0.96/89), whereas *S.funingense* gen. et sp. nov. is positioned outside (PP/BV = 1/100). The pairwise distance based on the Kimura 2 parameter model showed that most of the pairwise genetic distances between the 12 species beyond a threshold of 0.04 (Table 1). The genetic distances between *Songpotamon* gen. nov. and the other genera ranged from 0.042 to 0.178, and the minimum value within *Songpotamon* gen. nov. was 0.042. Considering the profound morphological differences and the genus/species level genetic divergence among these genera and species, we revealed that *Songpotamon* gen. nov. and the two new species of this new genus, indeed represent distinct taxa of Potamidae.

**Table 1:** Matrix of pairwise nucleotide divergences based on the Kimura 2 parameter model using 16S rDNA sequences between the species of *Songpotamon* gen. nov., *Parvuspotamon*, *Cantopotamon*, *Chinapotamon*, *Diyutamon*, and *Qianguimon*.

	1	2	3	4	5	6	7	8	9	10	11
1. *Parvuspotamonyuxiense*											
2. *Cantopotamonhengqinense*	0.135										
3. *Cantopotamonshangchuanense*	0.148	0.059									
4. *Chinapotamonlonglinense*	0.162	0.073	0.080								
5. *Chinapotamondepressum*	0.159	0.073	0.088	0.006							
6. *Chinapotamonglabrum*	0.165	0.064	0.083	0.026	0.028						
7. *Diyutamoncereum*	0.148	0.062	0.074	0.053	0.055	0.046					
8. *Qianguimonelongatum*	0.156	0.068	0.075	0.080	0.082	0.080	0.069				
9. *Qianguimonsplendidum*	0.154	0.080	0.085	0.090	0.092	0.085	0.067	0.041			
10. *Songpotamondixuense* comb. nov.	0.163	0.067	0.088	0.062	0.065	0.044	0.042	0.078	0.081		
11. *Songpotamonmalipoense* gen. et sp. nov.	0.160	0.068	0.090	0.066	0.068	0.052	0.044	0.084	0.084	0.042	
12. *Songpotamonfuningense* gen. et sp. nov.	0.178	0.083	0.097	0.084	0.087	0.068	0.074	0.098	0.104	0.054	0.060

Geographically, the three species of *Songpotamon* gen. nov. occur in two different drainages, with *S.dixuense* comb. nov. occurring in the Tuoniang River (tributaries of the Pearl River), and the two new species in the Panlong River and Nanli River, respectively (tributaries of Yuanjiang-Red River) (Fig. 1). Previous studies of stream-associated freshwater crabs have shown that drainage systems can drive species divergence and biogeographical patterns (Daniels et al. 2015; Fang et al. 2015; Shi et al. 2021). In the present study, we inferred the two hydrologic systems may contributed the genetic divergence between *S.dixuense* comb. nov. and the two new species of *Songpotamon* gen. nov. Meanwhile, the species divergence between two new species may be attributed to local adaptive evolution in different tributaries.

## Supplementary Material

XML Treatment for Parvuspotamon

XML Treatment for Parvuspotamon yuxiense

XML Treatment for Songpotamon

XML Treatment for Songpotamon funingense

XML Treatment for Songpotamon malipoense

## ﻿Additional information

### Author ORCIDs

Boyang Shi https://orcid.org/0000-0003-3306-3764

Da Pan https://orcid.org/0000-0001-5445-6423

Hongying Sun https://orcid.org/0000-0003-2311-1814

### Data availability

All of the data that support the findings of this study are available in the main text.
